# Usual Blood Pressure and Risk of New-Onset Diabetes: Evidence From 4.1 Million Adults and a Meta-Analysis of Prospective Studies

**DOI:** 10.1016/j.jacc.2015.07.059

**Published:** 2015-10-06

**Authors:** Connor A. Emdin, Simon G. Anderson, Mark Woodward, Kazem Rahimi

**Affiliations:** ∗ The George Institute for Global Health, University of Oxford, Oxford, United Kingdom; † Cardiovascular Research Group, Institute of Cardiovascular Sciences, University of Manchester, Manchester, United Kingdom; ‡ The George Institute for Global Health, University of Sydney, Sydney, Australia; § Division of Cardiovascular Medicine, Radcliffe Department of Medicine, University of Oxford, Oxford, United Kingdom

**Keywords:** body mass index, meta-analysis, regression dilution bias, BMI, body mass index, BNF, British National Formulary, BP, blood pressure, CPRD, Clinical Research Practice Datalink, DBP, diastolic blood pressure, HR, hazard ratio, RAS, renin-angiotensin system, SBP, systolic blood pressure

## Abstract

**Background:**

Reliable quantification of the association between blood pressure (BP) and risk of type 2 diabetes is lacking.

**Objectives:**

This study sought to determine the association between usual BP and risk of diabetes, overall and by participant characteristics.

**Methods:**

A cohort of 4.1 million adults, free of diabetes and cardiovascular disease, was identified using validated linked electronic health records. Analyses were complemented by a meta-analysis of prospective studies that reported relative risks of new-onset diabetes per unit of systolic blood pressure (SBP).

**Results:**

Among the overall cohort, 20 mm Hg higher SBP and 10 mm Hg higher diastolic BP were associated with a 58% and a 52% higher risk of new-onset diabetes (hazard ratio: 1.58; 95% confidence interval [CI]: 1.56 to 1.59; and hazard ratio: 1.52; 95% confidence interval: 1.51 to 1.54), respectively. There was no evidence of a nadir to a baseline BP of 110/70 mm Hg. The strength of the association per 20 mm Hg higher SBP declined with age and with increasing body mass index. Estimates were similar even after excluding individuals prescribed antihypertensive or lipid-lowering therapies. Systematic review identified 30 studies with 285,664 participants and 17,388 incident diabetes events. The pooled relative risk of diabetes for a 20 mm Hg higher usual SBP across these studies was 1.77 (1.53 to 2.05).

**Conclusions:**

People with elevated BP are at increased risk of diabetes. The strength of the association declined with increasing body mass index and age. Further research should determine if the observed risk is modifiable.

In 2011, type 2 diabetes affected 366 million people worldwide; this prevalence is estimated to increase to 552 million by 2030 (1). Individuals with type 2 diabetes are at increased risk of major cardiovascular events, including ischemic heart disease, stroke, and heart failure (2). In a contemporary analysis of a U.K. primary care population, type 2 diabetes was associated with twice the risk of all-cause mortality and 3 times the risk of cardiovascular mortality relative to age- and sex-matched controls (3). Consequently, prevention of diabetes is critically important for reducing the burden of cardiovascular disease.

Although hypertension has long been recognized as an independent risk factor for fatal and nonfatal vascular events (4), the relationship between blood pressure (BP) and risk of new-onset diabetes is less clear. Elevated BP is associated with chronic inflammation (5) and endothelial dysfunction (6), both of which appear to be mediators of diabetes risk 7, 8. There is, therefore, a biological rationale to suspect that elevated BP may cause new-onset diabetes. However, among 30 cohort studies that have reported the association of BP and diabetes, 12 concluded that no such association is apparent, whereas the others reported a considerably variable strength of association (Online Table 1). Moreover, even the largest previous cohorts have had limited power to investigate whether any observed positive association between BP and diabetes varied significantly by important patient features (9).

A detailed understanding of BP as a potential risk factor for diabetes will help us better understand and communicate risks with patients and can lead to more targeted prevention and management. We therefore undertook both an analysis of 4.1 million individuals free from diabetes and cardiovascular disease in a contemporary U.K. primary care population and a meta-analysis of existing prospective studies to reliably determine the association between BP and diabetes.

## Methods

We used prospectively collected records from the U.K. Clinical Practice Research Datalink (CPRD) to assemble a cohort of 4.1 million patients free from vascular disease and diabetes. An electronic health record system, covering approximately 9% of the U.K. population, CPRD has been validated for epidemiological research into a range of diagnoses 10, 11. Eligible patients were additionally linked to Hospital Episode Statistics for secondary care/hospitalization data and to cause-specific mortality data.

### Participants, exposures, and outcomes

Patients were eligible for inclusion if they had a BP measurement performed between January 1, 1990, and January 1, 2013, and were between 30 and 90 years (inclusive) of age at the time of measurement. Additionally, patients needed to have their age recorded and be registered at a general practice for at least 1 year. To reduce measurement error to which single BP measurements are prone and to diminish the impact of short-term fluctuations in BP on observed associations, the initial measurement was transformed into “usual blood pressure” to adjust for regression dilution bias and the calculated usual BP was used as the exposure. All patients with pre-existing vascular disease (ischemic heart disease, cerebrovascular disease, heart failure, peripheral vascular disease, or renal disease) and diabetes (either type 1 or type 2) were excluded. Baseline covariates (body mass index [BMI], total cholesterol, high-density lipoprotein cholesterol, and smoking status) were defined as the closest measurement within 2 years of the baseline BP measurement for that covariate.

The primary outcome was a diagnosis of type 2 diabetes, defined as either clinical diagnosis of type 2 diabetes or diabetes unspecified (because 90% of diabetes cases are type 2 [12]) or prescription of insulin/antidiabetic drugs, as defined in the British National Formulary (BNF) chapters 6.1.1 and 6.1.2. Participants were censored at the earliest occurrence of the primary outcome, transfer out of practice, death, or last collection date of practice.

### Statistical analyses

Cox models, stratified by practice to account for clustering at the practice level, were used to determine hazard ratios (HRs) for BP categories for each outcome. The proportional hazards assumption was tested by plotting Schoenfeld residuals. The primary analysis was adjusted for age, sex, BMI, smoking status, baseline antihypertensive use (BNF chapters 2.2.1, 2.2.3, 2.2.4, 2.4, 2.5, 2.6.2), and baseline lipid-lowering agent use (BNF chapter 2.12), although further adjustment was undertaken in sensitivity analyses. Blood pressure was analyzed both as a continuous variable (per 20/10 mm Hg higher BP) and as a categorical variable. Usual systolic blood pressure (SBP) was defined by category: ≤95 mm Hg, >195 mm Hg, and increments of 10 mm Hg for everything in between (e.g., 96 to 105 mm Hg, 106 to 115 mm Hg, and so on). Usual diastolic blood pressure (DBP) also was defined by the measured diastolic BP categories: ≤65 mm Hg, >115 mm Hg, and for 10 mm Hg increments for everything in between (e.g., 66 to 75 mm Hg, and so on). BP categories were entered simultaneously into the Cox model (separate models for SBPs and DBPs) and estimated simultaneously. Floating absolute risks were used to display HRs for BP categories because floating absolute risks do not require the selection of a baseline group for display of standard errors (13). The variance of each estimate approximates the variance in the underlying category.

Multiple imputation using chained equations was used to impute missing covariates; 5 imputations were generated.

Measurement error and short-term fluctuations in BP will bias any potential association of BP with an outcome of interest to the null (an effect termed regression dilution bias). Because we were interested in the etiological association of BP with risk of new-onset diabetes, free of regression dilution bias, we used similar methods to the Emerging Risk Factors Collaboration to adjust for regression dilution bias 14, 15, 16, 17. That is, we regressed serial BP measurements within the median follow-up on the baseline BP measurement, but used generalized estimating equations rather than linear models to account for multiple serial BP measurements among participants. Regression dilution ratios were calculated as the inverse of the coefficient relating the serial measurements to the baseline measurement. Regression dilution ratios of 2.1 for SBP and 2.5 for DBP were estimated. Continuous HRs for measured BP (i.e., per 20/10 mm Hg) were then multiplied by these ratios to estimate the association for usual BP. For example, if an HR for baseline SBP of 1.5 was calculated, the HR for usual SBP was calculated as: exp (2.1·log[1.5]) = 2.3. For displaying HRs of BP as a categorical variable (i.e., 120 to 130 mm Hg measured SBP), measured BP was “shrunk” toward the overall mean BP by the calculated regression dilution ratios, as performed previously (18). For instance, if the overall mean of the baseline SBP measurements was 130 mm Hg and the mean of a specific BP category was 140, the mean usual BP of that category was calculated as: (140 mm Hg − 130 mm Hg)/2.1) + 130 = 135 mm Hg.

Six sensitivity analyses were conducted. First, models were further adjusted for total and high-density lipoprotein cholesterol. Second, models were further adjusted for year of the initial BP measurement, as a categorical variable (1990 to 1994, 1995 to 1999, 2000 to 2004, 2005 to 2009, and 2010 to 2013) to control for potential cohort effects. Third and fourth, to minimize the risk of reverse causality, individuals with an event in the first 2 years of follow-up and an event in the fourth year of follow-up were excluded. Fifth, individuals prescribed antihypertensive medication or lipid-lowering drugs at baseline or during follow-up were excluded because statins and classes of antihypertensive agents have been associated with an increased risk of diabetes 19, 20. Sixth, diagnosis of diabetes was defined as an explicit diagnosis of type 2 diabetes (that is, excluding individuals who were only diagnosed with unspecified diabetes or prescribed antidiabetic medicine without a concomitant diagnosis of type 2 diabetes).

### Meta-analysis

A systematic search strategy was designed and conducted by an experienced research librarian to identify previous studies of the association between BP and risk of new-onset diabetes. The strategy included MeSH terms and synonyms for the terms blood pressure, incident, and diabetes. MEDLINE was searched for articles published between 1966 and January 2015, with no language restrictions applied. Prospective observational studies, including observational analyses of randomized trials, were eligible for inclusion if they: 1) had at least 1 year of follow-up; 2) were reported a risk per unit of SBP that could be standardized to 20 mm Hg higher SBP; and 3) were adjusted for, at minimum, sex, age, and BMI. The latter was required for adjustment given that BMI is a strong risk factor for diabetes (21) and is associated with elevated BP (22). Studies conducted in populations immediately after renal transplantation and studies that examined gestational diabetes were excluded. Measures of relative risks (e.g., HRs and odds ratios), difference in BP, study population, number of incident diabetes events, and degree of adjustment were extracted in duplicate. Because no study adjusted for regression dilution bias, we pooled relative risks standardized to 20 mm Hg higher usual SBP by multiplying the reported relative risks per 20 mm Hg higher measured SBP by our regression dilution coefficient. Random effects meta-analysis was used due to the presence of high heterogeneity (I^2^ >50%). Test for interaction between subgroups was performed using Cochran’s Q test.

One study reported a relative risk of new-onset diabetes per 10 mm Hg higher DBP; this was assumed to correspond to a relative risk per 20 mm Hg higher SBP. Five studies reported relative risks of new diabetes comparing one-fifth of BP relative to another fifth but did not explicitly report a difference in SBP between fifths 9, 21, 23, 24, 25. A normal approximation was therefore assumed to determine the approximate difference in BP between fifths to allow for standardization per 20 mm Hg higher SBP. These 5 studies were excluded in a sensitivity analysis.

Analyses were performed using R, version 3.0 (R Foundation for Statistical Computing, Vienna, Austria).

## Results

A total of 4,694,120 individuals in the CPRD cohort had at least 1 BP measurement with at least 1 year of follow-up and were between 30 and 90 years of age, inclusive. After excluding 195,623 patients with existing diabetes and an additional 366,359 patients who had pre-existing cardiovascular disease, we identified a cohort of 4,132,138 individuals (Online Figure 1).

At baseline, women comprised 2,310,268 (55.9%) of the participants (Table 1). The median age was 46 years (interquartile range: 36 to 59), whereas the median BMI, before imputation, was 25.7 kg/m^2^ (interquartile range: 23.0 to 29.2 kg/m^2^). During a median follow-up of 6.8 years, 186,698 new-onset diabetes events were observed.

**Table 1:** Baseline Characteristics

	Baseline Usual BP∗			Overall (n = 4,132,138)
	<127 mm Hg (n = 1,474,462)	127-136 mm Hg (n = 1,541,802)	>136 mm Hg (n = 1,115,874)	
Follow-up, yrs	6.4 (2.7-11.1)	6.7 (3.0-11.0)	7.3 (3.4-11.2)	6.8 (3.0-11.1)
Age at baseline, yrs	39 (33-48)	46 (37-57)	59 (48-70)	46 (36-59)
Women	983,263 (66.7)	771,273 (50.0)	555,732 (49.8)	2,310,268 (55.9)
BMI, kg/m^2^†	24.3 (21.9-27.3)	26.2 (23.5-29.6)	27.3 (24.4-30.9)	25.7 (23.0-29.2)
Smoking status†				
Current smoker	369,263 (30.1)	356,553 (28.4)	218,099 (25.2)	943,915 (28.2)
Never smoker	671,681 (54.7)	677,155 (53.9)	469,610 (54.2)	1,818,446 (54.3)
Former smoker	186,428 (15.2)	223,496 (17.8)	179,436 (20.7)	589,360 (17.6)
Cholesterol (mmol/l)†				
Total	5.2 (4.6-6.0)	5.5 (4.8-6.2)	5.7 (5.0-6.4)	5.5 (4.8-6.3)
HDL	1.4 (1.1-1.7)	1.4 (1.2-1.7)	1.3 (1.1-1.6)	1.4 (1.1-1.7)
Most deprived fifth	275,902 (18.7)	286,718 (18.6)	217,095 (19.5)	779,715 (18.9)
Medication usage				
Antihypertensive at baseline	53,248 (3.6)	124,596 (8.1)	212,781 (19.1)	390,625 (9.5)
Antihypertensive during follow-up	191,575 (13.0)	381,426 (24.7)	581,966 (52.2)	1,154,967 (28.0)
Lipid-lowering at baseline	10,037 (0.7)	24,215 (1.6)	24,604 (2.2)	58,856 (1.4)
Lipid-lowering during follow-up	98,305 (6.7)	214,055 (13.9)	280,189 (25.1)	592,549 (14.3)

Values are median (interquartile range) or n (%).

BMI = body mass index; BP = blood pressure; HDL = high-density lipoprotein.

∗ Baseline usual BP categories of <127 mm Hg, 127 to 136 mm Hg, and >136 mm Hg correspond to baseline measured BP categories of <121 mm Hg, 121 to 140 mm Hg, and >140 mm Hg.

† Proportion of variables missing: BMI (31.2%), smoking status (18.9%), total cholesterol (74.6%), HDL cholesterol (81.3%). No other variables contained missing values.

### Associations of usual BP with new-onset diabetes

**Figure 1:**
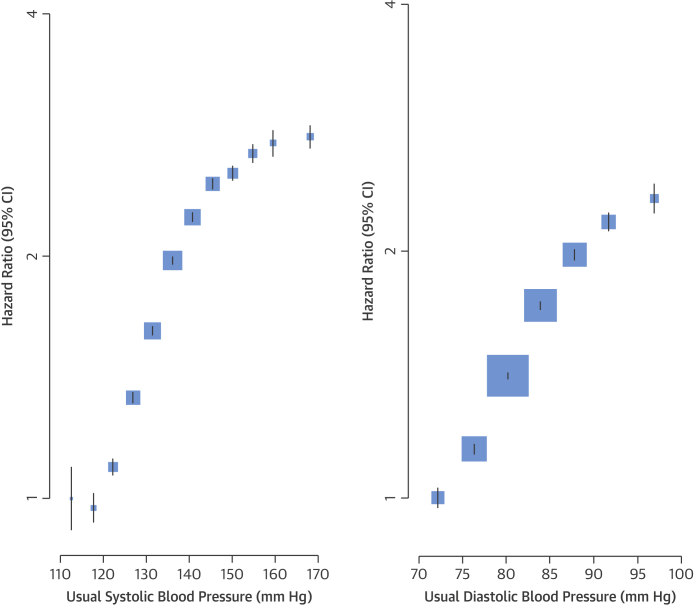
Adjusted HR for Diabetes by Systolic Blood Pressure and Diastolic Blood Pressure Hazard ratios for diabetes rose with increasing blood pressure. Adjustments were made for age, body mass index, smoking status, sex, and blood pressure category (plotted). CIs are displayed as floating absolute risks. **Area of each square** is proportional to the inverse variance of the estimate. Usual SBP categories were defined by the measured SBP categories: <95 mm Hg, >195 mm Hg, and increments of 10 mm Hg for everything in between (e.g., 95 to 105 mm Hg, 106 to 115 mm Hg, and so on); usual DBP categories were defined by the measured DBP categories: <65 mm Hg, >115 mm Hg, and intervening increments of 10 mm Hg (66 to 75 mm Hg, and so on). Blood pressure categories were entered simultaneously into the Cox model (separate models for SBP and DBP) and estimated simultaneously. Floating absolute risks were used to display all hazard ratios (13). The variance of each estimate approximates the variance in the underlying category. CI = confidence interval.

When analyzed using Kaplan-Meier curves, individuals with elevated BP had greater incidence of new diabetes during follow-up (Central Illustration). When analyzed using Cox proportional hazards models, adjustment for age, sex, and BMI attenuated diabetes risk (unadjusted HR: 2.60; 95% CI: 2.58 to 2.62 per 20 mm Hg higher SBP; adjusted HR: 1.58; 95% CI: 1.57 to 1.60 per 20 mm Hg higher SBP), although additional adjustment for smoking, antihypertensive therapy use, and lipid-lowering therapy use had little effect on estimates (Central Illustration). Usual SBP was continuously related to risk of new-onset diabetes (Figure 1). Although there was no evidence of a nadir down to a usual SBP level of 110 mm Hg, a flattening of the curve was observed below approximately 120 mm Hg. The association was strongest among individuals with a normal to mildly elevated BP, with an additional flattening of the curve above 150 mm Hg. Usual DBP was also continuously related to risk of new diabetes, with no evidence of a nadir or plateau in the range from 70 to 100 mm Hg. Overall, a 20 mm Hg higher SBP and a 10 mm Hg higher DBP were associated with a 58% increase (HR: 1.58; 95% CI: 1.56 to 1.59) and 52% increase (HR: 1.52; 95% CI: 1.51 to 1.54) in risk of newly diagnosed diabetes, respectively.

**Central Illustration:**
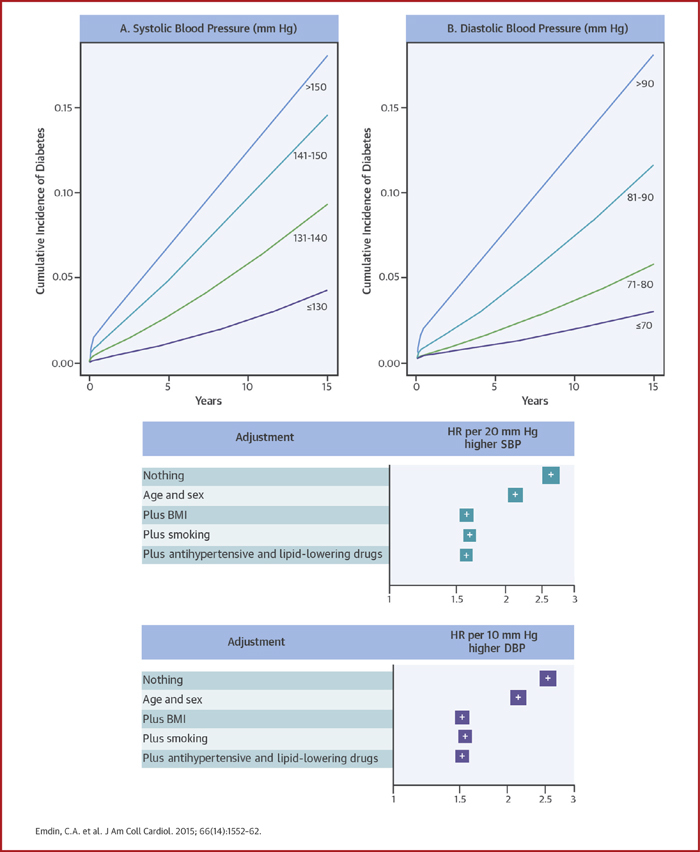
Elevated Blood Pressure and Risk of New-Onset Diabetes **(Top)** Kaplan-Meier curves illustrating time to incident diabetes by **(A)** usual systolic blood pressure categories and **(B)** usual diastolic blood pressure categories. No adjustments were applied. **(Bottom)** Hazard ratios per 20 mm Hg higher systolic blood pressure and 10 mm Hg higher diastolic blood pressure for new-onset diabetes, with progressive adjustment for age, sex, BMI, smoking and baseline antihypertensive use and baseline lipid-lowering drug use. BMI = body mass index; CI = confidence interval; DBP = diastolic blood pressure; HR = hazard ratio; SBP = systolic blood pressure.

**Figure 2:**
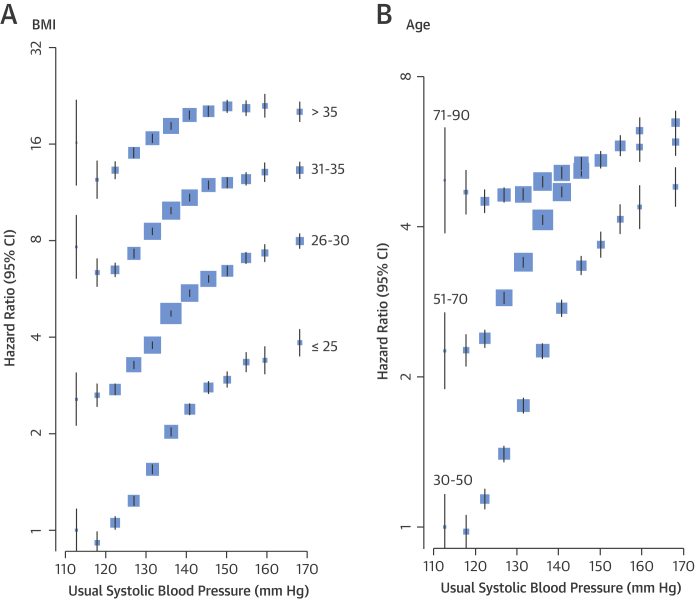
Adjusted Hazard Ratios for Diabetes by SBP and BMI or Age Adjustments were for smoking status, sex, and the interaction between SBP as a categorical variable and **(A)** BMI category (plotted) and **(B)** age category (plotted). BMI = body mass index; other abbreviations as in Figure 1.

**Figure 3:**
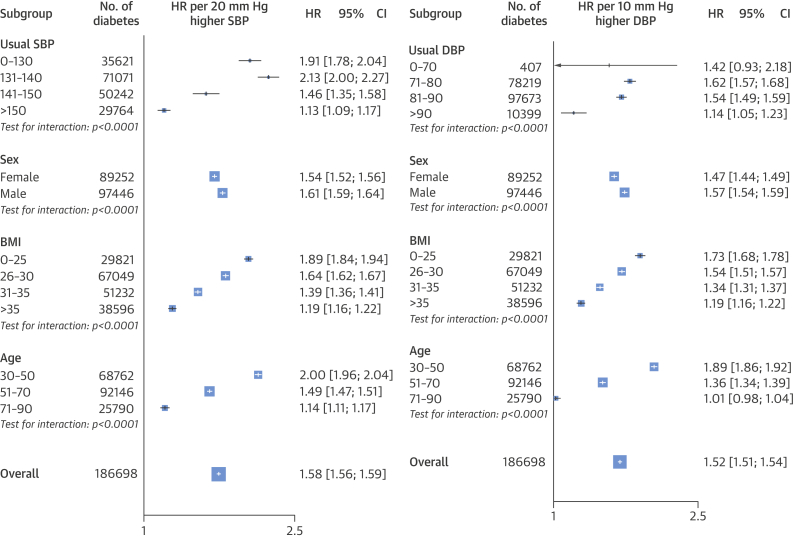
Association Between Blood Pressure and Diabetes per Baseline Variables Adjustments were for age, sex, BMI, baseline antihypertensive use, and baseline lipid-lowering therapy use. For subgroups of age, adjustment was also for age category and the interaction between SBP and age category (plotted). For subgroups of sex, adjustment was also for the interaction between sex and SBP (plotted). For subgroups of BMI, adjustments were also for BMI category and the interaction between SBP and BMI category (plotted). **Area of each square** is proportional to the inverse variance of the estimate. In various subgroups (usual SBP or DBP, sex, BMI, and age), proportional associations were seen between newly diagnosed diabetes and a 20 mm Hg higher SBP or 10 mm Hg higher DBP. A greater proportional risk was seen with the lowest BMI category versus the highest in both blood pressure groups; similarly, increasing age was associated with decreasing risk. Abbreviations as in Figures 1 and 2.

The proportional association between SBP and newly diagnosed diabetes differed by baseline BMI and age (Figures 2 and 3). A 20 mm Hg higher SBP was associated with a greater proportional increase in the risk of diabetes among individuals with a BMI ≤25 kg/m^2^ (HR: 1.89; 95% CI: 1.84 to 1.94) than among individuals with a BMI >35 kg/m^2^ (HR: 1.19; 95% CI: 1.16 to 1.22; p for interaction < 0.0001). However, the higher baseline absolute risk at BMI >35 kg/m^2^ resulted in larger absolute risk increases in diabetes per 20 mm Hg higher SBP in this BMI range (Figure 2). A 10 mm Hg higher usual DBP was associated with a 73% higher risk of diabetes among individuals with a BMI <20 kg/m^2^ (HR: 1.73; 95% CI: 1.68 to 1.78), whereas it was associated with a 19% higher risk among individuals with a BMI >35 kg/m^2^ (HR: 1.19; CI: 1.16 to 1.22; p for interaction: < 0.0001) (Figure 3).

The relative risk of new-onset diabetes per 20 mm Hg higher usual SBP declined with increasing age, from an HR of 2.00 (95% CI: 1.96 to 2.04) at 30 to 50 years of age to an HR of 1.14 (95% CI: 1.11 to 1.17) at 71 to 90 years of age (Figures 2 and 3). However, because of the increasing absolute risk of diabetes with older age, the absolute risk differences per 20 mm Hg usual SBP were similar across the different age groups, despite the declining relative risks. Risk of diabetes per 10 mm Hg higher DBP similarly declined with increasing age, from an HR of 1.89 (95% CI: 1.86 to 1.92) to 1.01 (95% CI: 0.98 to 1.04) (Figure 3).

When SBP and DBP was included in the same model, both were positively related to risk of diabetes with similar strengths of association; 20 mm Hg usual SBP was associated with an HR of 1.42 (95% CI: 1.40 to 1.44) and 10 mm Hg usual DBP was associated with an HR of 1.51 (95% CI: 1.49 to 1.54).

When all individuals prescribed BP-lowering drugs or statins at baseline or during follow-up were excluded in a sensitivity analysis, estimates were similar (Online Figure 2). Further adjustment for total and high-density lipoprotein cholesterol, socioeconomic status, and period of initial BP measurement also had little effect on estimated associations between SBP and DBP and risk of new-onset diabetes (Online Figures 3 and 4). Exclusion of individuals diagnosed with diabetes within the first 2 years and within the first 4 years of follow-up also had little effect on associations (Online Figures 5 and 6). Estimates also did not change materially when diabetes was restricted to explicit diagnosis of type 2 diabetes (Online Figure 7).

### Meta-analysis

**Figure 4:**
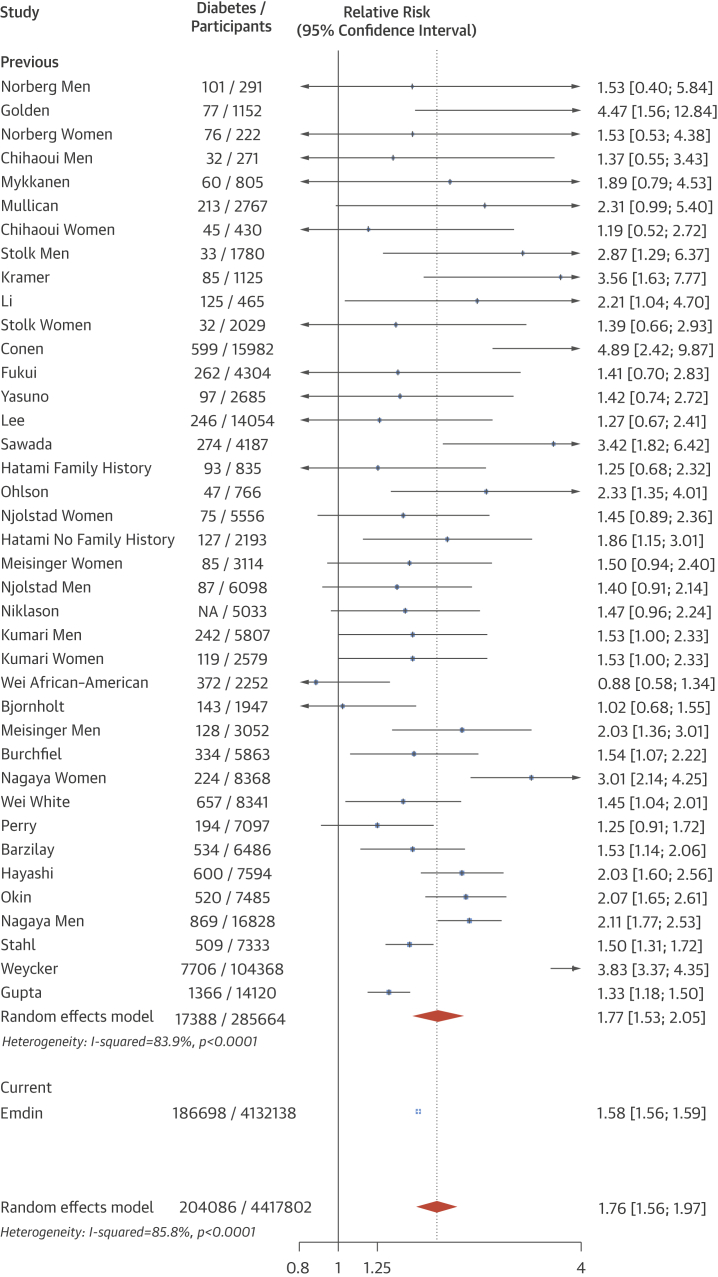
Association Between Higher Usual SBP and Diabetes Risk A meta-analysis showed that each 20 mm Hg higher usual SBP from previous cohort studies was associated with a 77% higher risk of new diabetes. Study refers to first author of study. Abbreviations as in Figures 1 and 2.

Thirty prospective observational studies were identified (Online Figure 8), with 285,664 participants and 17,388 incident diabetes events. Pooled random effects meta-analysis showed that each 20 mm Hg higher usual SBP from prior cohort studies was associated with a 77% higher risk of new diabetes (relative risk: 1.77; 95% CI: 1.53 to 2.05) (Figure 4). A test for interaction with our estimate of 58% higher risk of diabetes per 20 mm Hg usual SBP was not significant (p = 0.14). The overall pooled coefficient, including our and previous cohort studies, was 76% per 20 mm Hg higher usual SBP (relative risk: 1.76; 95% CI: 1.56 to 1.97). Estimates were similar when 5 studies that used a normal approximation to determine the BP difference associated with the provided relative risk were excluded (Online Figure 9).

## Discussion

In a cohort of people without known previous vascular disease and with more than 180,000 presentations of newly diagnosed diabetes, both SBP and DBP were found to continuously relate to risk of new-onset diabetes, with no evidence of a nadir down to 110/70 mm Hg. The strengths of associations varied substantially between different subpopulations. In particular, relative risks per 20 mm Hg higher SBP and 10 mm Hg higher DBP declined with increasing age and with increasing BMI. Nevertheless, because of higher absolute risk of diabetes at older age and with higher BMI, a 20 mm Hg difference in SBP and 10 mm Hg difference in DBP were still associated with substantial absolute risk differences in old age and overweight.

Previous observational analyses have been conflicted on the relationship between elevated BP and diabetes risk (Online Table 1). For example, in an observational analysis of the Losartan Intervention For Endpoint study, 20 mm Hg higher SBP was associated with a 39% higher risk of new diabetes (HR: 1.39; 95% CI: 1.25 to 1.56) (26); in the Women’s Health Study, hypertensive individuals had twice the risk of developing diabetes relative to those with SBP between 120 and 129 mm Hg (HR: 2.03; 95% CI: 1.77 to 2.32) (9). Additionally, higher on-treatment SBP was associated with a higher risk of new-onset diabetes in the International Verapamil SR-Trandolapril study (27). However, in a prospective cohort study of risk factors for diabetes in 7,097 men, no association was observed between baseline BP and diabetes risk after adjustment for clinical and demographic covariates (21). These qualitatively and quantitatively discrepant findings are most likely because of the limited power of individual studies to reliably measure modest risk associations, as supported by our systematic review, which shows that the pooled estimate across these studies support a modest association between elevated SBP and risk of diabetes (Figure 4).

Our results support the hypothesis that elevated BP is associated with increased risk of diabetes. Indeed, our estimate of a 58% increase in risk of new-onset diabetes per 20 mm Hg higher usual SBP did not differ significantly from a pooled estimate. In addition to confirming the overall effects from previous reports, our study’s large sample size extends previous reports by investigating the differences in associations by key population characteristics, such as age, sex, and BMI (Central Illustration). The importance of these more detailed subpopulation analyses is highlighted by the large differences in the relative risks by patient characteristics in our cohort. For example, in contrast with a previous report, in which no interaction by BMI (on the basis of 1,672 events) was observed, we found that a 20 mm Hg higher SBP was associated with an 89% higher risk among individuals with a BMI ≤25 kg/m^2^, but a 19% higher risk among people with a BMI >35 kg/m^2^.

It is unclear whether the observed association between BP and diabetes is causal. The Nateglinide and Valsartan in Impaired Glucose Tolerance Outcomes Research trial, which supports a causal relationship, randomized 9,306 patients to valsartan or placebo and established a 2.8 mm Hg SBP difference between arms. Incidence of diabetes in the valsartan arm was significantly reduced (HR: 0.86; 95% CI: 0.80 to 0.92) (28). However, a network meta-analysis of randomized trials of antihypertensive medication observed that only angiotensin-converting enzyme inhibitors and angiotensin receptor blockers reduced diabetes risk (20), suggesting it is renin-angiotensin system (RAS) activation that is causally related to risk of new-onset diabetes and not BP per se. However, the lack of a reduction in new diabetes observed for diuretics, beta-blockers, and calcium-channel blockers may be due to off-target effects for these therapies rather than a lack of a relationship between BP and diabetes risk (29). Previous analyses have suggested that a causal relationship between mediators of chronic inflammation, specifically interleukin-6, and incident diabetes may exist 7, 30. Chronic inflammation characterizes both obesity (31) and elevated BP (5), risk factors for diabetes, and is reduced by RAS inhibition (32). Thus, chronic inflammation may mediate, in part, the relationship between both risk factors (obesity and hypertension) and incident diabetes. Alternatively, endothelial dysfunction may link elevated BP and diabetes (8). An individual patient data meta-analysis, such as the Blood Pressure Lowering Treatment Trialists’ Collaboration, would be ideally suited to examine whether BP lowering, independent of RAS inhibition, reduces the risk of new diabetes.

Assuming causality, this analysis suggests that individual- and population-based efforts to lower BP may also lower the incidence of diabetes. Because RAS inhibition has been demonstrated to reduce the incidence of new diabetes in randomized trials, prescription of angiotensin-converting enzyme inhibitors and angiotensin receptor blockers has the most reliable evidence base for reducing the incidence of diabetes at an individual level. Although we observed a declining proportional association between BP and risk of diabetes with increasing BMI, the greater absolute risk of diabetes at higher BMI would support targeting individuals with high BMI for BP lowering to prevent diabetes. Population-based efforts to lower BP, for example, by reducing alcohol consumption through policy interventions or by promoting exercise, may also lead to reductions in the incidence of diabetes. However, further research is needed to examine the causality of the described associations and determine whether BP lowering without renin-angiotensin inhibition would reduce risk of new-onset diabetes.

### Study strengths and limitations

This analysis has several strengths, including its large size, encompassing more than 4.1 million individuals and 180,000 incident diabetes events, and contemporary nature. One potential limitation is that we used routinely collected electronic health records for our analysis. Although this approach has recently been used to examine the relationship between type 2 diabetes and cardiovascular risk, it is possible that some of our type 2 diabetes events may have been misclassified (e.g., metabolic syndrome but not type 2 diabetes). However, a previous study suggested that physician-diagnosed type 2 diabetes events in CPRD are highly reliable (33). Furthermore, we supplemented our analysis with a meta-analysis of prospective observational studies within 285,664 individuals and 17,388 incident diabetes events. This complementary approach increased our study’s reliability, allowing us to first validate our estimate against previous studies and then examine the relationship between BP and risk of diabetes in various subpopulations. Our estimate of an overall 58% increase per 20 mm Hg higher usual SBP was consistent with our meta-analysis of previous observational studies that largely used adjudicated diabetes events and was consistent in 6 sensitivity analyses, including an analysis of explicit diagnoses of type 2 diabetes (rather than unspecified diabetes).

## Conclusions

A 20 mm Hg higher SBP was associated with a 58% higher risk of new-onset diabetes, whereas a 10 mm Hg higher DBP was associated with a 52% higher risk of developing diabetes. The strength of the association declined with increasing BMI and age. Further investigation is needed to determine whether this association is causal.

Perspectives **COMPETENCY IN MEDICAL KNOWLEDGE:** Elevated blood pressure is associated with the risk of developing diabetes, and there is no nadir in the normotensive range.

**TRANSLATIONAL OUTLOOK:** Randomized trials are needed to establish causation and to determine whether lowering blood pressure, particularly by administration of inhibitors of the renin-angiotensin-aldosterone system, will reduce the risk of developing diabetes.
