# Supplementary material for: Usual Blood Pressure and Risk of New-Onset Diabetes: Evidence From 4.1 Million Adults and a Meta-Analysis of Prospective Studies
Source: J Am Coll Cardiol. 2015 Oct 6;66(14):1552–62. doi: 10.1016/j.jacc.2015.07.059 (PMC4595710; doi:10.1016/j.jacc.2015.07.059)
Supplement: Online Table 1 and Online Figures 1–9 [file mmc1.docx]

**Supplementary Appendix**

Online Table 1. Previous analyses of the relationship between blood pressure and risk of new onset diabetes.

| **Study (Author, Year)** | **Name of Cohort(s)** | **Location** | **Baseline Year** | **Population** | **Participants** | **Incident Diabetes** | **Adjustments** | **Median Follow Up (Years)** | **Was a significant association between BP and diabetes observed?** |
| --- | --- | --- | --- | --- | --- | --- | --- | --- | --- |
| ***Previous cohort studies*** | | |  |  |  |  |  |  |  |
| Ohlson, 1988(1) | Gothenburg 1913 Cohort | Gothenburg, Sweden | 1967 | General population, men born in 1913 | 766 | 47 | Adjusted for BMI, family history of diabetes, other clinical/demographic variables | 13.5 | Yes |
| Stolk, 1993(2) | Zoetermeer | Zoetermeer, Netherlands | 1975-1978 | Men from general population | 3809 | 65 | Stratified by sex, adjustment for age, BMI | 11.5 | Yes for men, no for women |
| Li, 1994(3) | Daqing (Li) | Daqing, China | 1986 | General population | 465 | 125 | Age, BMI, sex, fasting glucose | 6 | Yes |
| Mykkanen, 1994(4) | Kuopio | Kuopio, Finland | 1986-1988 | Elderly subjects aged 65-74 | 805 | 60 | Age, sex, BMI, 2-h insulin and other clinical/demographic variables | 3.5 | No |
| Burchfiel, 1995(5) | Honolulu Heart Program 45-54 | Hawaii, United States | 1965-1968 | Men from general population | 5863 | 334 | Age, BMI, exercise, other clinical/demographic characteristics (restricted to men) | 6 | Yes |
| Perry, 1995(6) | British Regional Heart Study | United Kingdom | 1978-1980 | Men from general population | 7097 | 194 | Age, BMI, exercise, other clinical/demographic characteristics | 12.8 | No |
| Njolstad, 1998(7) | Finnmark | Finnmark, Norway | 1977-1978 | General population | 11654 | 162 | Age, BMI, cholesterol, other clinical/demographic characteristics | 12 | No |
| Hayashi, 1999(8) | Osaka Health Survey | Osaka, Japan | 1981-1991 | Male employees of a gas company | 7594 | 600 | Age, BMI, alcohol consumption, smoking habit, other clinical/demographic variables | 9.6 | Yes |
| Chihaoui, 2000(9) | Tunis | Tunis, Tunisia | 1985 | General population | 701 | 77 | Stratification by age, adjustment for BMI, age, family history of diabetes, other clinical/demographic variables | 10 | No |
| Bjornhol, 2001(10) | Oslo | Oslo, Norway | 1972 | Healthy men | 1947 | 143 | Age, BMI, family history of diabetes, other clinical/demographic variables | 22.5 | No |
| Golden, 2003(11) | Johns Hopkins Precursor Study | Baltimore, United States | 1948-1964 | Male former medical students | 1152 | 77 | BMI, family history of diabetes, smoking, physical activity | 38 | Yes |
| Kumari, 2004(12) | Whitehall II | London, United Kingdom | 1985-1988 | Men from general population | 8386 | 361 | Stratified by sex, adjusted for age, BMI, other demographic/clinical characteristics | 10.5 | No |
| Conen, 2007(13) | Women's Health Study | United States | 1993 | Female health professionals | 15982 | 599 | Age, BMI, ethnicity, alcohol consumption, family history of diabetes, other clinical and demographic characteristics | 10.2 | Yes |
| Norberg, 2007(14) | Vasterbotten Health Study | Vasterblotten, Sweden | 1989-2000 | General population | 513 | 177 | Stratification by sex, adjustment for age, BMI, glucose and other clinical/demographic variables | 8.8 | No |
| Meisinger, 2008(15) | MONICA Augsburg | Augsburg, Germany | 1984-1985/1989-1990/1994-1995 | General population | 6166 | 213 | Stratified by sex, adjusted for age, survey and BMI | 7.6 | Yes |
| Mullican, 2009(16) | San Antonio Heart Study | San Antonio, United States | 1979-1988 | Mexican Americans and non-Hispanic Whites in General Population | 2767 | 213 | Age, sex, BMI, ethnicity, family history of diabetes, other clinical/demographic characteristics | 7.8 | No |
| Weycker, 2009(17) | Kaiser | United States | 1998 | General population | 104368 | 7706 | Age, sex, BMI | 5 | Yes |
| Hatami, 2010(18) | Tehran Lipid and Glucose | Tehran, Iran | 1999-2001 | Women from general population | 3028 | 220 | Stratification by family history of diabetes, adjustment for age, BMI, other demographic/clinical variables | 6.4 | Yes with no family history of diabetes, no with family history of diabetes |
| Kramer, 2010(19) | Rancho Bernardo | California, United States | 1984-1987 | Middle-class Caucasian adults | 1125 | 85 | Age, sex, BMI, family history of diabetes, physical activity | 8.3 | Yes |
| Lee, 2010(20) | Seoul | Seoul, Korea | 2003-2008 | General population | 14054 | 246 | Age, sex, BMI and fasting glucose | 5 | No |
| Nagaya, 2010(21) | Nagoya | Nagoya, Japan | 1988-1991 | General population without disease | 25196 | 1093 | Matched for age, adjusted for BMI, cholesterol and other clinical/demographic variables | 8.2 | Yes |
| Sawada, 2010(22) | Tokyo Gas Company | Tokyo, Japan | 1985 | Men from gas company | 4187 | 274 | Age, BMI, alcohol, smoking, other clinical/demographic variables | 14 | Yes |
| Fukui, 2011(23) | Kyoto | Kyoto, Japan | 1998-2003 | General population | 4304 | 262 | Age, sex, BMI, cholesterol, other clinical/demographic variables | 5 | No |
| Wei, 2011(24) | ARIC/CARDIA/Framingham | United States | 1987-1989/1985-1986/1971-1975 | General population | 10593 | 1029 | Age, sex, BMI, cholesterol, other clinical/demographic variables | 8.9 | Yes |
| Stahl, 2012(25) | Gothenburg Primary Prevention Study | Gothenburg, Sweden | 1970-1973 | Men from general population | 7333 | 509 | Age, BMI, antihypertensive treatment, cholesterol, other demographic/clinical variables | 28 | Yes |
| ***Previous observational analyses of randomized trials*** | | | |  |  |  |  |  |  |
| Niklason, 2004(26) | CAPPP | Sweden and Finland | 1990-1994 | Untreated hypertensive individuals | 5033 | NA | Age, BMI, other clinical characteristics (sex not independently associated) | 6.1 | No |
| Barzilay, 2006(27) | ALLHAT | United States/Canada | 1994-1998 | Hypertension, coronary heart disease risk factor | 6486 | 534 | Age, sex, ethnicity, BMI, randomized treatment, other clinical/demographic variables | 2 | Yes |
| Gupta, 2008(28) | ASCOT | UK/Ireland/Nordic countries | 1998-2000 | Hypertensive individuals with cardiovascular risk factors | 14120 | 1366 | Age, sex, BMI, randomized treatment, other clinical/demographic characteristics | 5.5 | Yes |
| Yasuno, 2010(29) | CASE-J | Japan | 2001-2002 | High cardiovascular risk hypertensive patients | 2685 | 97 | Age, sex, BMI, antihypertensive use, other demographic/clinical variables | 3.3 | No |
| Okin, 2013(30) | LIFE | United States, Europe | 1995-1997 | Hypertension and left ventricular hypertrophy | 7485 | 520 | Age, sex, BMI, antihypertensive use, other demographic/clinical variables | 4.7 | Yes |
| ***Current study*** | | |  |  |  |  |  |  |  |
| Emdin, 2015 | CPRD | United Kingdom | 1990-2012 | General population, free of vascular disease | 4132138 | 186698 | Age, BMI, smoking status, other clinical/demographic characteristics | 6.8 | Yes |

366 359 individuals had:

- A history of cardiovascular disease

4 132 138 individuals with 186 698 diagnoses of diabetes:

- 160 243 diagnoses of type 2 diabetes
- 26 455 diagnoses of unspecified diabetes

15 236 931 individuals in CPRD (January 2014)

4 694 120 individuals remaining

10 542 811 were not:

- between the ages of 30 and 90, inclusive, at baseline
- had a BP measurement
- had at least one year of follow up in CPRD

195 623 individuals had either a:

- Prior diagnosis of diabetes
- Prescription of an antidiabetic drug

4 498 497 individuals remaining

Online Figure 1.

Online Figure 2. Adjusted hazard ratios per 20 mm Hg higher systolic blood pressure by blood pressure, sex, BMI and age. Adjustments were for age, sex, BMI, baseline antihypertensive use and baseline lipid use. For subgroups of age, adjustment was also for age category and the interaction between systolic BP and age category (plotted). For subgroups of sex, adjustment was also for the interaction between sex and systolic BP (plotted). For subgroups of BMI, adjustments were also for BMI category and the interaction between systolic BP and BMI category (plotted). All individuals prescribed lipid-lowering drugs or antihypertensives at baseline or at any time during follow up are excluded. Area of each square is proportional to the inverse variance of the estimate.

Online Figure 3. Adjusted hazard ratios per 20 mm Hg higher systolic blood pressure by blood pressure, sex, BMI and age. Adjustments were for age, sex, BMI, baseline antihypertensive use, baseline lipid use, total cholesterol and HDL cholesterol. For subgroups of age, adjustment was also for age category and the interaction between systolic BP and age category (plotted). For subgroups of sex, adjustment was also for the interaction between sex and systolic BP (plotted). For subgroups of BMI, adjustments were also for BMI category and the interaction between systolic BP and BMI category (plotted). Area of each square is proportional to the inverse variance of the estimate.

Online Figure 4. Adjusted hazard ratios per 20 mm Hg higher systolic blood pressure by blood pressure, sex, BMI and age. Adjustments were for age, sex, BMI, baseline antihypertensive use, baseline lipid use, total cholesterol, HDL cholesterol and period of blood pressure measurement (1990-1994, 1995-1999, 2000-2004, 2005-2009, 2010-2013). For subgroups of age, adjustment was also for age category and the interaction between systolic BP and age category (plotted). For subgroups of sex, adjustment was also for the interaction between sex and systolic BP (plotted). For subgroups of BMI, adjustments were also for BMI category and the interaction between systolic BP and BMI category (plotted). Area of each square is proportional to the inverse variance of the estimate.

Online Figure 5. Adjusted hazard ratios per 20 mm Hg higher systolic blood pressure by blood pressure, sex, BMI and age. Adjustments were for age, sex, BMI, baseline antihypertensive use and baseline lipid use. For subgroups of age, adjustment was also for age category and the interaction between systolic BP and age category (plotted). For subgroups of sex, adjustment was also for the interaction between sex and systolic BP (plotted). For subgroups of BMI, adjustments were also for BMI category and the interaction between systolic BP and BMI category (plotted). First two years of follow up are excluded. Area of each square is proportional to the inverse variance of the estimate.

Online Figure 6. Adjusted hazard ratios per 20 mm Hg higher systolic blood pressure by blood pressure, sex, BMI and age. Adjustments were for age, sex, BMI, baseline antihypertensive use and baseline lipid use. For subgroups of age, adjustment was also for age category and the interaction between systolic BP and age category (plotted). For subgroups of sex, adjustment was also for the interaction between sex and systolic BP (plotted). For subgroups of BMI, adjustments were also for BMI category and the interaction between systolic BP and BMI category (plotted). First four years of follow up are excluded. Area of each square is proportional to the inverse variance of the estimate.

Online Figure 7. Adjusted hazard ratios per 20 mm Hg higher systolic blood pressure by blood pressure, sex, BMI and age. Adjustments were for age, sex, BMI, baseline antihypertensive use and baseline lipid use. For subgroups of age, adjustment was also for age category and the interaction between systolic BP and age category (plotted). For subgroups of sex, adjustment was also for the interaction between sex and systolic BP (plotted). For subgroups of BMI, adjustments were also for BMI category and the interaction between systolic BP and BMI category (plotted). Diabetes diagnoses are restricted to explicit diagnoses of type 2 diabetes, with prescription of antidiabetic drugs and diagnoses of unspecified diabetes excluded. Area of each square is proportional to the inverse variance of the estimate.

4516 studies identified and screened:

- 4512 studies identified from MEDLINE Search
- 4 studies identified from bibliographic review

143 studies excluded:

- 7 did not adjust or stratify by age, BMI and sex
- 17 could not be standardized per 20 mm Hg higher SBP
- 11 were a duplicated cohort
- 96 did not report an association by blood pressure
- 11 were not prospective studies
- 1 was after kidney transplantation

173 studies screened in full text review.

4343 studies excluded during initial screen for violating inclusion criteria:

- Unrelated population or outcome
- Did not adjust or stratify by age, BMI and sex
- Not an observational analysis

30 prospective studies identified

Online Figure 8. Flowchart of study identification.

15910 / 251834

202608 / 4383972

Online Figure 9. Association between a 20 mm Hg higher usual systolic blood pressure and risk of diabetes. Five trials for which a normal approximation was used to standardize reported relative risks to a difference in blood pressure were excluded. Study refers to first author of study (characteristics provided in Online Table 1).

Online Supplementary Appendix References

1. Ohlson LO, Larsson B, Björntorp P, et al. Risk factors for type 2 (non-insulin-dependent) diabetes mellitus. Thirteen and one-half years of follow-up of the participants in a study of Swedish men born in 1913. Diabetologia 1988;31:798–805.

2. Stolk RP, van Splunder IP, Schouten JS, Witteman JC, Hofman A, Grobbee DE. High blood pressure and the incidence of non-insulin dependent diabetes mellitus: findings in a 11.5 year follow-up study in The Netherlands. Eur. J. Epidemiol. 1993;9:134–139.

3. Li GW, Zhang H, Hu YH. [Essential hypertension: a predictor of the 6 year-incidence of NIDDM in 465 non-diabetics]. Zhonghua Nei Ke Za Zhi 1994;33:654–657.

4. Mykkänen L, Kuusisto J, Pyörälä K, Laakso M, Haffner SM. Increased risk of non-insulin-dependent diabetes mellitus in elderly hypertensive subjects. J. Hypertens. 1994;12:1425–1432.

5. Burchfiel CM, Curb JD, Rodriguez BL, et al. Incidence and predictors of diabetes in Japanese-American men. The Honolulu Heart Program. Ann Epidemiol 1995;5:33–43.

6. Perry IJ, Wannamethee SG, Walker MK, Thomson AG, Whincup PH, Shaper AG. Prospective study of risk factors for development of non-insulin dependent diabetes in middle aged British men. BMJ 1995;310:560–564.

7. Njølstad I, Arnesen E, Lund-Larsen PG. Sex differences in risk factors for clinical diabetes mellitus in a general population: a 12-year follow-up of the Finnmark Study. American journal of epidemiology 1998;147:49–58.

8. Hayashi T, Tsumura K, Suematsu C, Endo G, Fujii S, Okada K. High normal blood pressure, hypertension, and the risk of type 2 diabetes in Japanese men. The Osaka Health Survey. Diabetes care 1999;22:1683–1687.

9. Chihaoui M, Kanoun F, Ben Rehaiem B, et al. Predictive risk factors for deterioration from normoglycemic state to type 2 diabetes mellitus or impaired glucose tolerance in a Tunisian urban population. Diabetes Metab. 2001;27:487–495.

10. Bjørnholt JV, Erikssen G, Liestøl K, Jervell J, Erikssen J, Thaulow E. Prediction of Type 2 diabetes in healthy middle-aged men with special emphasis on glucose homeostasis. Results from 22.5 years' follow-up. Diabet. Med. 2001;18:261–267.

11. Golden SH, Wang N-Y, Klag MJ, Meoni LA, Brancati FL. Blood pressure in young adulthood and the risk of type 2 diabetes in middle age. Diabetes care 2003;26:1110–1115.

12. Kumari M, Head J, Marmot M. Prospective Study of Social and Other Risk Factors for Incidence of Type 2 Diabetes in the Whitehall II Study. Archives of internal medicine 2004;164:1873.

13. Conen D, Ridker PM, Mora S, Buring JE, Glynn RJ. Blood pressure and risk of developing type 2 diabetes mellitus: the Women's Health Study. Eur. Heart J. 2007;28:2937–2943.

14. Norberg M, Stenlund H, Lindahl B, et al. Components of Metabolic Syndrome Predicting Diabetes: No Role of Inflammation or Dyslipidemia*. Obesity 2007;15:1875–1885.

15. Meisinger C, Thorand B, Schneider A, Stieber J, Döring A, Löwel H. Sex differences in risk factors for incident type 2 diabetes mellitus: the MONICA Augsburg cohort study. Archives of internal medicine 2002;162:82–89.

16. Mullican DR, Lorenzo C, Haffner SM. Is Prehypertension a Risk Factor for the Development of Type 2 Diabetes? Diabetes care 2009;32:1870–1872.

17. Weycker D, Nichols GA, O'Keeffe-Rosetti M, et al. Excess risk of diabetes in persons with hypertension. Journal of Diabetes and its Complications 2009;23:330–336.

18. Hatami M, Hadaegh F, Khalili D, Sheikholeslami F, Azizi F. Family history of diabetes modifies the effect of blood pressure for incident diabetes in Middle Eastern women: Tehran Lipid and Glucose Study. Journal of human hypertension 2011;26:84–90.

19. Kramer CK, Mühlen DV, Barrett-Connor E. Mid-life blood pressure levels and the 8-year incidence of type 2 diabetes mellitus: the Rancho Bernardo Study. Journal of human hypertension 2009;24:519–524.

20. Lee WY, Kwon CH, Rhee EJ, et al. The effect of body mass index and fasting glucose on the relationship between blood pressure and incident diabetes mellitus: a 5-year follow-up study. Hypertens. Res. 2011;34:1093–1097.

21. Nagaya T, Yoshida H, Takahashi H, Kawai M. Resting heart rate and blood pressure, independent of each other, proportionally raise the risk for type-2 diabetes mellitus. Int J Epidemiol 2010;39:215–222.

22. Sawada SS, Lee IM, Naito H, et al. Long-Term Trends in Cardiorespiratory Fitness and the Incidence of Type 2 Diabetes. Diabetes care 2010;33:1353–1357.

23. Fukui M, Tanaka M, Toda H, et al. Risk factors for development of diabetes mellitus, hypertension and dyslipidemia. Diabetes Res. Clin. Pract. 2011;94:e15–e18.

24. Wei GS, Coady SA, Goff DC, et al. Blood pressure and the risk of developing diabetes in african americans and whites: ARIC, CARDIA, and the framingham heart study. Diabetes care 2011;34:873–879.

25. Stahl C, Novak M, Lappas G, et al. High-normal blood pressure and long-term risk of type 2 diabetes: 35-year prospective population based cohort study of men. BMC Cardiovasc Disord 2012;12:89.

26. Niklason A, Hedner T, Niskanen L, Lanke J, Group CPPS. Development of diabetes is retarded by ACE inhibition in hypertensive patients--a subanalysis of the Captopril Prevention Project (CAPPP). J. Hypertens. 2004;22:645–652.

27. Barzilay JI, Davis BR, Cutler JA, et al. Fasting glucose levels and incident diabetes mellitus in older nondiabetic adults randomized to receive 3 different classes of antihypertensive treatment: a report from the Antihypertensive and Lipid-Lowering Treatment to Prevent Heart Attack Trial (ALLHAT). Archives of internal medicine 2006;166:2191–2201.

28. Gupta AK, Dahlöf B, Dobson J, et al. Determinants of new-onset diabetes among 19,257 hypertensive patients randomized in the Anglo-Scandinavian Cardiac Outcomes Trial--Blood Pressure Lowering Arm and the relative influence of antihypertensive medication. Diabetes care 2008;31:982–988.

29. Yasuno S, Ueshima K, Oba K, et al. Is Pulse Pressure a Predictor of New-Onset Diabetes in High-Risk Hypertensive Patients?: A subanalysis of the Candesartan Antihypertensive Survival Evaluation in Japan (CASE-J) trial. Diabetes care 2010;33:1122–1127.

30. Okin PM, Hille DA, Wiik BP, et al. In-treatment HDL cholesterol levels and development of new diabetes mellitus in hypertensive patients: The LIFE Study. Diabet. Med. 2013;30:1189–1197.
